# Health system description and assessment: a scoping review of templates for systematic analyses

**DOI:** 10.1186/s12961-024-01166-y

**Published:** 2024-07-11

**Authors:** Ruth Waitzberg, Isabel Pfundstein, Anna Maresso, Bernd Rechel, Ewout van Ginneken, Wilm Quentin

**Affiliations:** 1 Department of Health Care Management, Faculty of Economics and Management, Technische Universität Berlin, Straße Des 17. Juni 135, 10623 Berlin, Germany; 2 Department of Health Care Management, Faculty of Economics and Management, Technische Universität Berlin, Berlin, Germany; 3 European Observatory on Health Systems and Policies, Berlin, Germany; 4 European Observatory on Health Systems and Policies, London School of Hygiene and Tropical Medicine, London, United Kingdom; 5 European Observatory on Health Systems and Policies, Department of Health Care Management, Technische Universität Berlin, Strasse Des 17. Juni 135, 10623 Berlin, Germany; 6 Planetary & Public Health, University of Bayreuth, Bayreuth, Germany; 7 German West-African Centre for Global Health and Pandemic Prevention, Department of Health Care Management, Technische Universität Berlin, Berlin, Germany

**Keywords:** Health policy, Health system design, Scoping review, Template

## Abstract

**Background:**

Understanding and comparing health systems is key for cross-country learning and health system strengthening. Templates help to develop standardised and coherent descriptions and assessments of health systems, which then allow meaningful analyses and comparisons. Our scoping review aims to provide an overview of existing templates, their content and the way data is presented.

**Main body:**

Based on the WHO building blocks framework, we defined templates as having (1) an overall framework, (2) a list of indicators or topics, and (3) instructions for authors, while covering (4) the design of the health system, (5) an assessment of health system performance, and (6) should cover the entire health system. We conducted a scoping review of grey literature published between 2000 and 2023 to identify templates. The content of the identified templates was screened, analyzed and compared. We found 12 documents that met our inclusion criteria. The building block `health financing´ is covered in all 12 templates; and many templates cover ´service delivery´ and ´health workforce’. Health system performance is frequently assessed with regard to ‘access and coverage’, ‘quality and safety’, and ‘financial protection’. Most templates do not cover ‘responsiveness’ and ‘efficiency’. Seven templates combine quantitative and qualitative data, three are mostly quantitative, and two are primarily qualitative. Templates cover data and information that is mostly relevant for specific groups of countries, e.g. a particular geographical region, or for high or for low and middle-income countries (LMICs). Templates for LMICs rely more on survey-based indicators than administrative data.

**Conclusions:**

This is the first scoping review of templates for standardized descriptions of health systems and assessments of their performance. The implications are that (1) templates can help analyze health systems across countries while accounting for context; (2) template-guided analyses of health systems could underpin national health policies, strategies, and plans; (3) organizations developing templates could learn from approaches of other templates; and (4) more research is needed on how to improve templates to better achieve their goals. Our findings provide an overview and help identify the most important aspects and topics to look at when comparing and analyzing health systems, and how data are commonly presented. The templates were created by organizations with different agendas and target audiences, and with different end products in mind. Comprehensive health systems analyses and comparisons require production of quantitative indicators and complementing them with qualitative information to build a holistic picture.

*Clinical Trial Registration*:  Not applicable.

**Supplementary Information:**

The online version contains supplementary material available at 10.1186/s12961-024-01166-y.

## Background

Health systems strengthening is key to achieving Sustainable Development Goal (SDG) 3 “to ensure healthy lives and promote well-being for all at all ages” [1], and is high up on the global research and policy agendas [2, 3]. Strengthening health systems requires a comprehensive approach targeting different health system building blocks (see Box 1) and a thorough understanding of the multiple relationships and interactions between them [4].

Describing and analysing the building blocks of health systems’ and their functioning is a precondition for assessing them, and an effective assessment supports health systems strengthening [5–8]. Utilizing a standardized format or template when describing and assessing health systems can support cross-country comparisons. This is because the reports that are produced based on templates follow the same structure, thus simplifying the extraction of comparable information and benchmarking [9–15], as well as the identification of high-performing areas of health systems and areas for improvement [16, 17]. Therefore, templates provide a basis for informed, evidence-based decision-making, promoting transparency, and facilitating cross-country learning and collaboration [18, 19]. Finally, standardized documents describing health systems support performance assessment by providing a common way of understanding health systems and their functioning, along with a set of indicators that allow for the comparison of health system performance across different countries. A number of international agencies have developed templates to describe and assess health systems and to facilitate international comparisons and ultimately highlight areas and policies for improvement [20, 21].

However, there are challenges associated with developing and maintaining standardized health system documents for cross-country comparison, as this exercise requires a common definition and understanding of the elements and functioning of health systems to be described, and a common way of describing, analyzing and interpreting these elements. It requires an inventory of consensual indicators that accurately capture and describe the elements and functions of health systems, that are reliable and available across countries [16, 17]. Effective templates that guide authors on how to write these standardized health system documents mitigate these challenges. Not only do templates guide authors on which elements and functions to describe, they also provide definitions and instructions on how to describe them, how to collect, present and interpret data and indicators, and how to compare one country to others. Yet, to our knowledge, an inventory of such templates does not exist, and a comparison of templates’ contents, strategies of guidance and list of indicators proposed is missing.

This paper fills these gaps by presenting a scoping review of templates that guide authors on how to analyze and compare health systems and to assess their performance. We assess templates against the WHO’s building blocks framework [22], which we take as foundational. Specifically, our objectives were (1) to identify existing templates that serve as guides for describing and analyzing health systems; and (2) to explore how the templates describe the design and performance of health systems, i.e. the methods of data collection and presentation and the topics covered.

## Materials and methods

A scoping review was conducted to identify existing templates. We conducted exploratory searches of templates between February and December 2020, which helped us conceptualize “what a template is” and what its defining elements are. This definition then informed the search strategy and the inclusion and exclusion criteria. We used Arksey and O’Malley [23] as guidance for our review stages (identify research question, identify relevant studies, study selection, charting data, reporting results). Because the tools and documents sought were grey literature, a systematic grey literature search approach was adopted following Godin et al. [24]. The protocol of this scoping review was registered with Open Science Framework (https://osf.io) and is accessible under the registration number 10.17605/OSF.IO/CVHJD. The review is reported according to the PRISMA-ScR extension for Scoping review guidelines [25].

### Conceptualization of “templates”

We identified six criteria that define a ‘template’ for the purpose of our analysis. These included: (1) the existence of an overall framework, (2) the provision of a list of indicators or clear descriptions of the topics and elements to be included, (3) instructions for authors on how to write an informative document, (4) guidance for the description of the design of the health system, and (5) an approach for the assessment of health system performance. In addition, (6) a “template” should cover the entire health system, which we operationalized as covering at least four of the six functions of WHO’s building blocks (see Box 1) [22].

### Data collection

A database search was conducted to identify templates from July to December 2023. Four information sources were identified as suggested by Godin [24]. First, four grey literature databases were identified (OpenGrey, ELDIS, WHOLIS, Google Scholar); second, a systematic search of the internet was conducted using Google, complemented by Open AI (BING AI, ELICIT). Third, websites from organizations that operate on an international level and whose agenda includes overall health or health systems were searched (e.g., WHO, OECD, World Bank). For a list of all targeted websites see supplement 1. Fourth, tacit knowledge of all authors, experts in health systems analysis, were integrated in the search.

A list of search terms (strings) was created initially based on the expertise of the co-authors and refined through a trial run. The search terms are presented in Table 1. Table 2 presents one example (out of many) of the search strategy. Due to the different characteristics of each database´s search engine mechanisms, different search strategies were applied.^1^ Only the first 100 hits were screened. All targeted websites were hand-searched. Full electronic search strategies are provided in Supplement 2.

**Table 1:** Search terms

Keywords	Search terms
1. Template	Report, assessment, tool, manual, guideline, guide, guidance, outline, evaluation, survey, profile, account, monitoring, description, instruction
2. Health system	Health system, health sector, health care, health care performance, health system performance, health
3. International	Cross-country, international, comparison, similarity, difference

**Table 2:** Search string for WHOLIS

Database	WHOLIS
Search Strings	1. (Report OR assessment OR tool OR manual OR guideline OR guide OR guidance OR outline OR evaluation OR survey OR profile OR account OR monitoring OR description OR instruction) AND (health system OR health sector OR health care system OR health care performance OR health) 2. 1 AND (cross-country OR international OR comparison OR similarity OR difference)
Filter	Search in: keywords; Publication date range: 2000–2023
Date of search	03.12.2023

#### Inclusion criteria

We searched primarily for templates, but also looked for reports that described health systems so we could search for the template that guided the report. Eligibility criteria for inclusion in the review were:

1. Documents that are used for health system description, comparison or performance assessment at the international level
2. Published between 2000 and 2023
3. Most current version of the document
4. Template must include six defining criteria
  - Include an overall framework
  - Include a list of indicators or clear descriptions that depict the topics and elements included
  - Include instructions for authors on how to write an informative document
  - Guide on how to describe the design of the health system
  - Include an approach for the assessment of the health system performance
  - Cover the entire health system, which we operationalized as covering at least four of the six functions of WHO’s building blocks

#### Exclusion criteria

1. National reports or templates—because these are not designed to fit multiple health systems.
2. Documents that focus only on specific aspects of health systems such as health for older adults, health inequalities.

Once a report met all inclusion criteria, except criterion 3 (Include instructions for authors), attempts were made to obtain the relevant template from the authors or publishing organizations. There were no language restrictions in the scoping review.

### Screening and extraction process

The screening process included three stages. First, the hits of each search were screened by title. Duplicates were removed and different issues of a report were condensed into one. Second, the introductions, abstracts and tables of contents were screened. Third, the full documents were assessed regarding the fulfilment of the inclusion criteria. Those that did, were defined as “templates” (Table 3). The third step was performed by two authors (IP and RW), and in case of divergent screening decisions, the reports in question were discussed with a third author (WQ).

### Data charting process and analysis

A data charting form was developed containing the templates name, their methods of data collection, building blocks and intermediate and final goals of the WHO’s framework. Two reviewers (RW, IP) charted data independently and discussed the results. Inconsistencies were discussed with a third author (WQ). For all identified “templates”, we matched the chapters of the documents to WHO’s building blocks framework to assess whether the tools covered at least four of the six building blocks. We assessed data collection approaches suggested by the templates, for example through interviews, surveys, literature review, administrative data and records (Table 4). In addition, we mapped chapters to the six intermediate and overall goals of the building blocks framework (Table 5). This was followed by an in-depth analysis of the templates.

Box 1 Definitions of WHO's six building blocks of a health system

**Table:** 

**Fig. 1:**
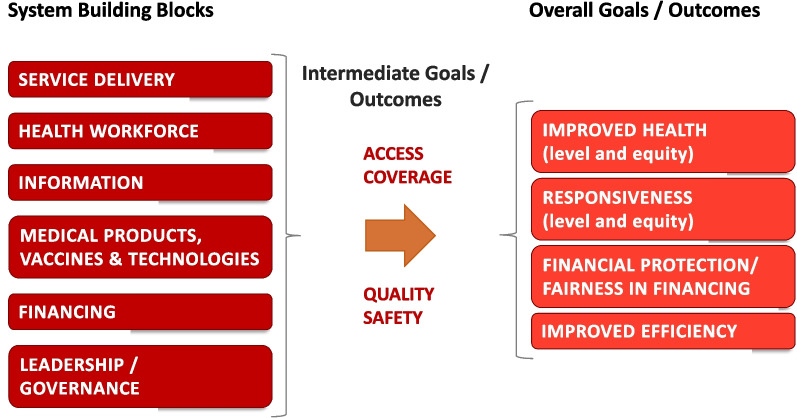
The WHO health system framework. Source: [22], with minor modifications

In 2007, the WHO developed a framework to guide countries’ health system strengthening efforts [22]. It defines a health system as “all organizations, people and actions whose primary intent is to promote, restore or maintain health”. In addition, the framework conceptualizes the health system in terms of six building blocks (see Fig. 1), including service delivery, health workforce, information, medical products and technologies, financing, and leadership/governance. Together, these building blocks contribute to achieving the intermediate goals of quality & safety and access & coverage, which in turn contribute to achieving the overall health system goals of improved health (level and equity), responsiveness (level and equity), social and financial risk-protection, and improved efficiency

## Results

### Identifying templates

**Fig. 2:**
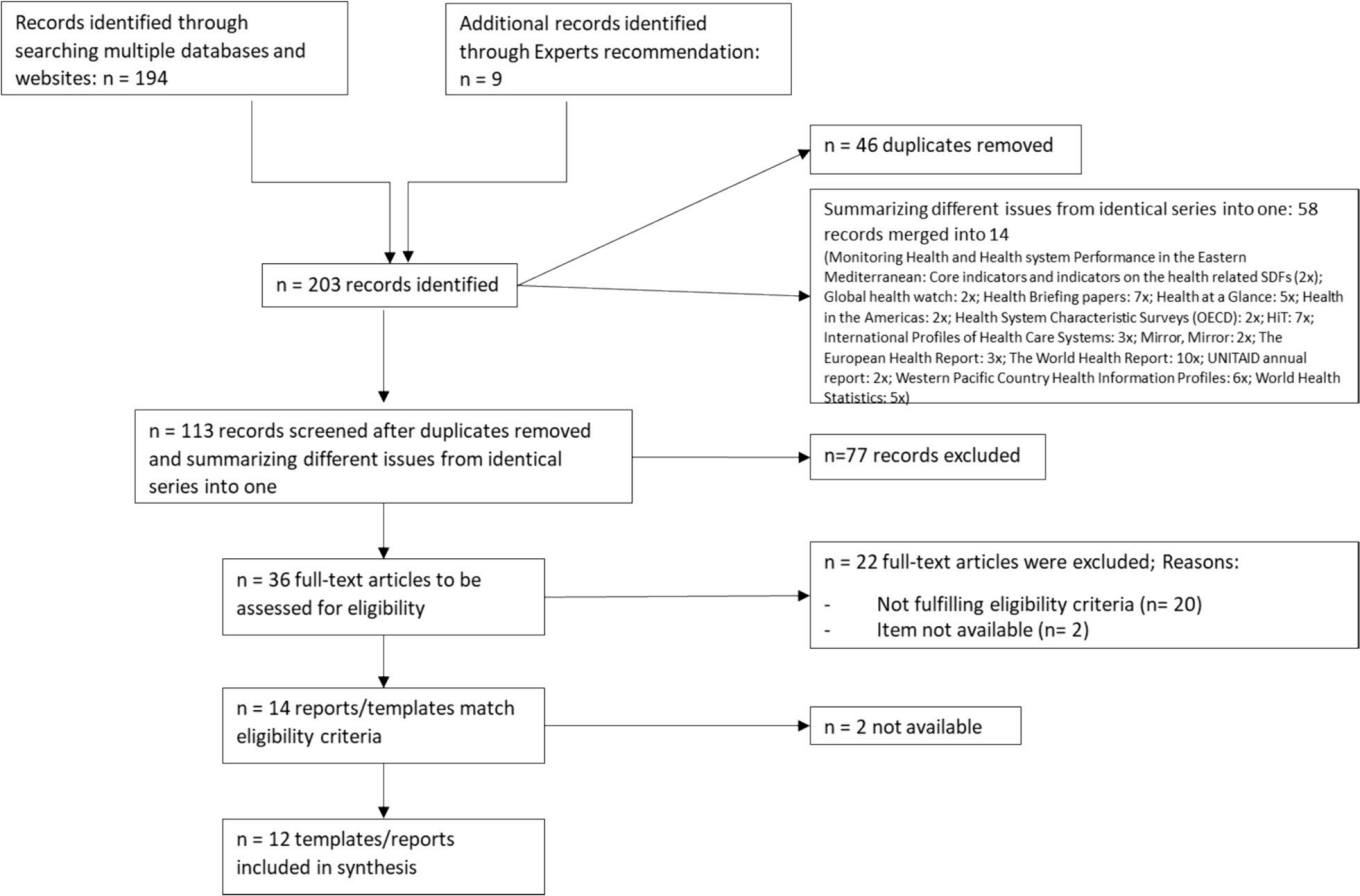
Illustration of the scoping review screening process

The scoping review identified 14 documents that met the inclusion criteria. Eight of these were publicly available templates. Six documents were reports, of which two could not be obtained from the authors (Country Health Profiles by EMRO; and Western Pacific Country Health Information Profiles). Ultimately, twelve templates could be included in the analysis. The screening process is illustrated in Fig. 2.

Table 3 presents the 12 templates identified. Another 26 documents met most of our criteria, but not all, or focused on specific topics (and not most building blocks), such as primary care; therefore, they were not considered “templates” (see Appendix 3 in the e-supplement). All tools and templates, with one exception (CEF-AHI), were developed by major organizations such as the WHO, the OECD, the USAID, the Commonwealth Fund, or collaborations between them.

**Table 3:** Identified documents and the extent to which they fulfill the criteria to be classified as templates

Document	Framework	List of indicators	Instructions for authors	System design/structure	System performance	Covers at least four of the six functions of WHO’s building blocks
Documents that cover several building blocks—considered ‘templates’						
1. A common evaluation framework for the African Health Initiative, 2013 CEF-AHI [26]	√	√	√	√	√	√
2. Commonwealth Fund health profiles (CMWF), 2020 [27]	√	√	√	√	√	√
3. AHOP Country Health Systems and Service Profile: An overview (CHSSP) [28]	√	√	√	√	√	√
4. OBS Health Systems in Transition (HiT) template for authors (HiT), 2019* [29]	√	√	√	√	√	√
5. Health Systems in Action insights. European Observatory on Health Systems and Policies. 2021 (HSiAI) [30]	√ (implicit)	√	√	√	√	√
6. Monitoring Framework for Universal Health in the Americas 2021. PAHO (MFUHA) [31]	√	√	√ (implicit)	√	√	√
7. OECD health systems characteristics survey (HSCS), Latin American Countries, 2018 [32, 33]	√ (implicit)	√	√ (minimal)	√	√	√
8. Pan American Health Organization (PAHO)/WHO/USAID Health Systems Country Profiles 1999–2009 (HSCP) [34]	√	√	√	√	√	√
9. State of Health in the EU country healthprofiles, (SoHEU) 2019 [35, 36]	√	√	√	√	√	√
10. USAID UHC Monitoring Framework, (UHC-MF) 2017 + Ethiopia country report [ 37 ]	√	√	√ (minimal)	√ (minimal)	√	√
12. USAID’s health system assessment approach: a how-to manual, Version 3.0 (HSAA) 2017 [38]	√	√	√	√	√	√
13. WHO monitoring the building blocks of health systems (WHO-MBB): a handbook of indicators and their measurement strategies, 2010 [39]	√	√	√	√	√	√

Notes: 1. These are the latest versions of the templates at time of search. By time of publication of this work, newer versions are available. Newer or older versions might have different contents, topics, indicators. 2. (minimal) = the element exists in the document, but only to a very limited extent. (*) There is a spinoff of the HiT template for authors (2010), that was adapted for the Asia Pacific region of the WHO in 2013 and 2016: https://apps.who.int/iris/bitstream/handle/10665/208276/9789290617570_eng.pdf?sequence=1. This adaptation does not add to the HiT template for authors, the approach to analyze and describe health systems was the very same, and due to its similarity, we do not consider it a separate template. 4. The criterion “framework” is marked as implicit in two templates. The HSiAI does not refer to a framework in its template, but since it is an offshoot of the HiT, it is implicitly based on a framework. For the HSCS, a clear framework is described in the corresponding working paper [40]

### Content of the templates

Table 4 provides an overview of data collection approaches suggested by the different templates. The OECD HSCS [32, 33] and the CMWF [27] are mostly qualitative, asking authors to answer closed and open-ended questions or suggesting in-depth interviews with stakeholders for data collection. Three templates, the CEF-AHI, WHO-MBB, and the USAID UHC-MF, are exclusively quantitative [26, 37, 39]. They focus only on quantitative indicators based on administrative data, censuses, and surveys. Seven templates, the HiT template [29], the SoHEU [36], the USAID HSAA [38], the PAHO HSCP [34], the CHSSP [28], the HSiAI [30], and the MFUHA [31] combine qualitative and quantitative approaches, describing and analyzing health systems with (qualitative) descriptions and (quantitative) indicators (see the first column of Table 4).

**Table 4:** Building blocks covered by the templates – chapter titles connected to the functions of health systems

Document	Methods of data collection	Building blocks covered					
		Service delivery	Health workforce	Health information systems	Medical products	Health system financing	Leadership and governance
A common evaluation framework for the African Health Initiative, 2013 CEF-AHI	Quant. Indicators from administrative data and primary data collection (surveys)		Inputs and Processes: Human resources		Inputs and Processes: Medicines, Equipment, Commodities	Inputs and Processes: Financing	Inputs and Processes: Governance and leadership
Commonwealth Fund health profiles (CMWF), 2020	Mostly qual. closed and open-ended questions to authors and a few quan. indicators from administrative data	How is the delivery system organized and how are providers paid?	Physician education and workforce	What is the status of electronic health records?	How are costs contained?	How does universal health coverage work?	How does universal health coverage work?
AHOP Country Health Systems and Service Profile: An overview (CHSSP)	Qual. open-ended questions; quant. indicators from administrative data and surveys	Service delivery	Health workforce	Health information and information systems		Health financing	Organization and governance of the health system
OBS Health Systems in Transition (HiT) template for authors (HiT), 2019*	Qual. open-ended questions; quant. indicators from administrative data and surveys	Provision of services	Physical and human resources; provision of services	Organization and governance, Physical and human resources	Financing, provision of services, Assessment of the health system	Financing	Organization and governance; Assessment of the health system
Health Systems in Action insights. European Observatory on Health Systems and Policies. 2021 (HSiAI)	Qual. open-ended questions; quant. indicators from administrative data and surveys	Generating resources, providing services, and ensuring access: Physical resources,	Generating resources, providing services, and ensuring access: human resources			Financing and ensuring financial protection: Health spending, Public and Private Spending	Organizing the health system: Organization and governance, Organization of health service provision,
Monitoring Framework for Universal Health in the Americas 2021. PAHO (MFUHA)	Qual. open-ended questions; quant. indicators from administrative data and surveys	Universal Health Output	Universal Health Output		Universal Health Output	Universal Health Output	
OECD health systems characteristics survey 2016 (HSCS)	Qual. closed and open-ended questions (and very few indicators)	Health care delivery	Governance and resource allocation, Health care delivery	Governance and resource allocation	Health care delivery	Health care financing	Governance and resource allocation
PAHO/WHO Health Systems Country Profiles 1999–2009 (HSCP)	Mostly Qual. closed and open-ended questions and a few quant. indicators from administrative data	Service provision	Steering role, Service provision		Service provision	Financing and assurance	Institutional mapping of the health system
State of Health in the EU country health profiles, (SoHEU) 2019	Qual. open-ended questions; quant. indicators from administrative data and surveys	The health system: organization, population coverage and health service delivery	The health system: physical and human resources			The health system: Health expenditure	The health system
USAID UHC Monitoring Framework, 2017 + Ethiopia country report (UHC-MF)	Quant. indicators from administrative data	Health Service Coverage	Health Service Coverage		Health Service Coverage	Financial Protection	
USAID’s the health system assessment approach: a how-to manual, 2017 (HSAA)	Qual. closed and open-ended questions, interviews with stakeholders and quant. indicators from administrative data and surveys	Country and health system overview; Service delivery	Human resources for health	Country and health system overview, Health information systems	Medical products, vaccines, and technologies	Country and health system overview; Health financing	Country and health system overview; Governance
WHO monitoring the building blocks of health systems: a handbook of indicators and their measurement strategies, 2010 (WHO-MBB)	Quant. routine administrative reporting data and records, census, surveys	Service delivery	Health workforce	Health information systems	Access to essential medicines	Financing	Leadership and governance

*Qual* qualitative, *Quan* quantitative

Table 4 also presents the health system functions covered by each template, indicating the names of the chapters where the topics are covered. Table 4 also shows that some functions, such as ‘service delivery’ and ‘financing’, are often covered in a separate, dedicated chapter. Other functions do not always have their own dedicated chapters, such as ‘health workforce’. Only the `Health Financing´ building block is covered in all the 12 templates, followed by ‘service delivery’ and ‘health workforce’, which are covered by 11 of the 12 templates. ‘Leadership and governance’ (10/12) and ‘medical products’ (7/12) follow in descending order. The building block least covered is Health information (5/12). The building block ‘medical products’ is almost exclusively covered by templates targeting middle- and low-income countries.

Table 5 presents the health system goals covered by each template. Two templates, i.e. the HiT, and the SoHEU, assess all health system goals. Eight templates consider most health system goals, while two templates, i.e. the WHO’s MBB and the USAID’s UHC-MF, focus only on a relatively narrow range of health system goals. The most frequently covered goals are ‘access and coverage’ (11/12) ‘quality and safety’ (10/12), and ‘financial protection’ (9/12). The other goals were covered at maximum by half of the templates (‘improved health’, 6/12). ‘Responsiveness’ and ‘efficiency’ are not covered by seven of the twelve templates.

**Table 5:** Goals covered by the templates—chapter titles connected to intermediate of final goals

Document	Access coverage	Quality and Safety	Improved health (level and equity)	Responsiveness	Social and financial risk protection	Improved efficiency	Other Topics covered
1. A common evaluation framework for the African Health Initiative, 2013 CEF-AHI	Outcomes: Coverage of interventions; Outputs: Intervention access & service readiness	Output: Intervention quality	Impact: Mortality and nutrition, Morbidity, Fertility				Equity
2. Commonwealth fund health profiles, 2020 (CMWF)	How does universal health coverage work? What is being done to reduce disparities?	What are the major strategies to ensure quality of care? What is being done to promote delivery system integration and care coordination?			What is being done to reduce disparities?	How are costs contained?	What major innovations and reforms have recently been introduced?
AHOP Country Health Systems and Service Profile: An overview (CHSSP)	Health service coverage and system outcomes: 10.2 Coverage of essential interventions; 10.1 Availability of essential services/// Performance of the health system- outputs: Access to essential services;	Health service coverage and system outcomes: 10.4 Health security// Performance of the health system- outputs: Quality of care in the provision of essential services		Health service coverage and system outcomes: 10.5 User satisfaction	Health service coverage and system outcomes: 10.3 Financial risk protection	Performance of the health system- outputs: 9.5 Health system efficiency	9.4 Resilience of the health system to sustain provision of essential services
OBS Health Systems in Transition (HiT) template for authors (HiT), 2019*	Assessment of the health system, financing	Assessment of the health system	Introduction	Assessment of the health system, Organization and governance	Assessment of the health system, Financing	Assessment of the health system	Socio-demographic and political context; principal health reforms
Health Systems in Action insights. European Observatory on Health Systems and Policies. 2021 (HSiAI)	Organizing the health system: Benefits packages and population coverage// Generating resources, providing services, and ensuring access: Provision of health services and accessibility		Improving the health of the population: Life expectancy, Infant and maternal mortality, Leading causes of death, risk factors		Financing and ensuring financial protection: Financial Protection,		Spotlight on antimicrobial resistance; European Programme of Work
Monitoring Framework for Universal Health in the Americas 2021. PAHO (MFUHA)	Universal Health Outcome	Universal Health Outcome	Universal Health Impact	Universal Health Outcome (% of pop reporting access barriers to health; % of women of reproductive age who have their need for family planning satisfied with modern methods)	Universal Health Outcome	-	
OECD health systems characteristics survey 2016 (HSCS)	Health care financing	Governance and resource allocation		Health care delivery	Health care financing		Provider payment mechanisms, competition, resource allocation
Pan American Health Organization (PAHO)/WHO/USAID Health Systems Country Profiles 1999–2009 (HSCP)	Steering role, Financing and assurance; Monitoring health systems change	Steering role, Service provision; Monitoring health systems change	Health situation analysis			Monitoring health systems change	Determinants of health; Monitoring health systems change
3. State of Health in the EU country health profiles, (SoHEU) 2019	The health system: Organization, population coverage, and health service delivery; Performance of the health system: accessibility, availability of services	Performance of the health system: effectiveness ( selected quality indicators),	Health Status; Mortality, Morbidity		Performance of the health system: accessibility ( population coverage, affordability),	Performance of the health system: effectiveness,	Resilience; Spotlight on mental health;
USAID UHC Monitoring Framework, (UHC-MF) 2017 + Ethiopia country report	Health Service Coverage, Financial Protection				Financial Protection		
USAID’s health system assessment approach: a how-to manual, Version 3.0 (HSAA) 2017	Service delivery	Service delivery	Country and health system overview	Service delivery	Service delivery		
WHO monitoring the building blocks of health systems (WHO-MBB): a handbook of indicators and their measurement strategies, 2010		Service delivery					

Most templates (eight of twelve) also cover topics beyond WHO’s building blocks framework. Some cover “extra topics” to contextualize the health system (e.g. socio-demographic and political context, and determinants of health), while others assess changes and reforms. Further topics covered in the templates are resilience; ageing and long-term care; provider payment mechanisms; equity, competition and resource allocation (see the last column of Table 5).

## Discussion

This is the first systematic scoping review of templates for standardized descriptions of health systems and for systematic assessments of performance. The review identified 12 templates, most of them written by international organizations. While most health system building blocks are addressed by all templates, some building blocks are less frequently covered, such as ‘medical products’ and ‘health information systems’. Similarly, some health system goals are not addressed by several templates, such as ‘responsiveness’ and ‘efficiency’. Seven templates provide guidance for collecting and presenting both quantitative and qualitative data, while three are mostly quantitative, and two are primarily qualitative. Our findings have implications for policy makers, for researchers and for organizations developing templates.

For policy makers, it is important to realize that health systems strengthening requires a systematic understanding of health systems. Many countries are developing strategic health system plans [41] and ‘health systems performance assessment’ strategies [5, 8, 42–46]. To assess health systems, policymakers and researchers require several tools that complement and build on each other. First, an analytical framework that defines the topics and aspects of health systems to be planned or assessed; second, descriptions of the structure and performance of the health system, based on the framework chosen; and third evaluations on the extent to which the system is performing and meeting its objectives [43]. While there are several analytical frameworks that define ‘a health system’ [5, 43, 46–49], and guides for health system performance assessments [5, 38, 42, 44, 50, 51], our work fills the knowledge gap in the second type of tools that describe the structures and functioning of the health system. First, by identifying the existing templates, second by analyzing their content, scope and methods, and third by highlighting how to improve these templates for a more systematic understanding of health systems structures and functioning.

Second, our work invites researchers to fill knowledge gaps. It calls for future research on a range of topics including: (1) identification of indicators ‘frequently used’ by templates, and their availability in different regions; (2) development of concepts and indicators for certain building blocks and health system goals (e.g. governance, health information, and responsiveness); and (3) the definition of “core” topics for templates. In addition, future work should explore whether templates achieve their goals of leading to standardized descriptions and assessments of health systems that are useful at the national level by informing policy-makers; and at the international level by facilitating health systems comparisons.

Finally, for organizations developing templates, our work might contribute to developing consensus towards elements that should be included in systematic analyses and assessments. In addition, results provide insights on three important aspects: (1) the relevance of context, (2) the potential to use more qualitative data for health systems analyses (3) and gaps of existing templates.

### Templates support standardized descriptions of health systems but need to reflect context

The templates identified in this study were created by organizations with different agendas and target audiences, and with different end products in mind, i.e. ranging from brief descriptions or assessments to full studies with detailed descriptions and assessments. Templates aimed at different groups of countries, either clustering them by region, such as the African Region [12, 26], the Americas [52], Europe [30, 36], or by income. About half of the templates correspond either to health systems of high-income countries (HICs) (SoHEU [36]) or low- and middle-income countries (LMICs) (CEF-AHI [26], MFUHA [31], AHOP [28], UHC-MF [37], HSAA [38], WHO-MBB [39]). Five templates have been used to guide descriptions of HIC and LMICs (CMWF [27], HiT [29], HSCS, HSCP [32, 33], HSiAI [30]).

The first reason for this is that organizations developing templates usually target specific regions, with unique needs and characteristics, or different income-level countries. Some organizations target either HICs or LMICs, but not both. For example, the OECD focuses primarily on HICs, whereas the WHO prioritized projects that promote LMICs. Certain organizations are region-specific and target a certain region. For example, PAHO targets Latin-American countries, AHOP and USAID target mainly African countries, and the European Observatory on Health Systems and Policies focusses on the European region.

Second, templates apparently cover data and information that is mostly relevant to these specific regions or either for HICs or LMICs. For example, while templates focusing on HICs cover diseases such as cancer and dementia, templates for LMICs focus on maternal and infant mortality, tropical diseases, and undernutrition [53–56]. While non-communicable diseases (NCDs) represent the main burden and mortality reasons also in regions such as Africa, NCDs are neglected in international development assistance and health system strengthening efforts, as is reflected in the templates. Templates focusing on Latin American countries address tropical diseases, while templates focusing on Europe focus on non-communicable and cardiovascular diseases. This finding raises the question of whether it is appropriate to cover different regions, LMIC and HIC systems in one comparison, given their idiosyncrasies. For example, LMICs rely more on traditional practitioners and healers [57–59] or receive more foreign donor funds than HICs [60, 61]. Organizations comparing and assessing health systems should take these differences into consideration, while attempting to compare the same health system functions and goals [62]. Third, templates that target HICs rely more on health service utilization and other routinely collected data that is less available in LMICs, while templates dedicated to LMICs generally rely on fewer indicators, and on survey-based indicators that are more commonly available for these countries [63]. These findings suggest that quantitative data and indicators are generally less available for LMICs [64–66], which calls for national and international agencies to improve and ensure the collection of robust data and health statistics as a precondition to describe, compare and assess the performance of health systems.

Nonetheless, templates should be generic enough to be suitable for all types of countries, allowing for adaptations to reflect context and particularities [43]. Resolving the apparent tension between standardising templates while reflecting context can be achieved by having multiple organizations with different target audiences compile a template jointly and agreeing on a common framework that suits different types of countries, and covers the topics to be covered within the health systems building blocks and goals, the methods of data collection, and the set of indicators proposed. Templates can achieve this dual objective by proposing a list of core elements and functions that must be described within each building block of the health system, and another list of elective topics that could be chosen based on the context of the country being described. While all countries should describe the essential elements of each building block and to what extent they achieve the ultimate goals, there can be additional aspects that are related to each building block, which are relevant only for specific contexts. For example, all countries should cover the building block ‘workforce’, and describe trends in density of nurses and physicians and geographical distribution as core topics. Yet, the focus on skill mix and the challenges faced by each country may differ, and can be described as elective topics that reflect context. As an elective topic, skill mix lends itself to context-specific elaboration: while HICs could focus on the balance between primary and specialist care, the role of physician assistants and specialist nurses, and explore concerns related to quality of workforce training, LMICs could highlight a more diverse range of professionals such as community workers, include traditional medicine in the analysis, and explore concerns regarding the shift of specialized workers to private practice. A third and related strategy consists of compiling a list of core indicators that support the description and analysis of core topics, and a list of elective indicators from which authors can choose how to describe the contextual factors. There has been extensive work on defining a list of ‘core’ indicators [16, 17], which can be further developed to define the set of indicators for the elective topics [18].

### There is an opportunity to use more qualitative data to analyze health systems

Existing macro-level health systems research has often focused on quantitative indicators, e.g. for classification of health systems into typologies [6, 62, 67, 68], for cross-country analyses of health reforms [69], or comparisons of performance [68, 70]. However, this data has several drawbacks: in high-income countries, there are frequent breaks in series and changing indicator definitions. In low-income countries, data on human and capital resources, service provision, and health system performance is often unavailable or outdated [63, 67, 81].

In several templates, qualitative descriptions are suggested to provide additional information on institutional or organizational health system characteristics, or explanations and interpretations of reported quantitative indicators, e.g. to explain breaks in series or discrepancies between national and international data. Qualitative information is more appropriate to capture processes, changes, and outcomes, and (over-)reliance on quantitative indicators may result in comparisons that are limited to quantifiable parameters. Therefore, comprehensive health systems analyses and comparisons require complementing quantitative indicators with qualitative information to build a more holistic picture [62, 64, 68]. If reporting of this information is sufficiently standardized, it can be useful for cross-country comparisons of health systems characteristics.

Qualitative data can be systematically collected from different sources including grey and academic literature, interviews with policymakers, scholars, health workers and managers, as well as interviews and focus groups among providers, patients and the general population. By using a standard interview guide, standard questionnaire, or table to be completed, data can be collected in a standardized manner that can be easily compared across countries. Qualitative data is particularly important when quantitative data or indicators are not collected, are not reliable or outdated. In addition, these sources can provide valuable information not captured by quantitative indicators, such as governance features, sources and content of information systems, or supply chains of pharmaceuticals. Interviews with stakeholders further shed light on processes that may complement or explain quantitative data, such as the considerations applied in health technology assessment beyond cost-effectiveness; (un)intended outcomes of payment mechanisms, such as transparency of financial flows, and additional parameters of quality of care that are not measured by quantitative indicators. Providers’, patients’ and the general population’s perspectives may add further value to quantitative indicators in understanding a host of issues, including quality of care, access barriers, dropout rates of health professions, or patient admission and treatment decisions.

### Health information systems, responsiveness and efficiency are missing in some templates

The building block ‘Health information’ is not always covered by existing templates, and the health system goals of ‘responsiveness’ and ‘efficiency’ are sometimes missing. The reasons for the relative lack of attention to these topics remain unclear [7, 11, 44, 71–74] but could be related to the lower availability (or absence) of quantitative indicators to describe and assess these topics in some of the countries. Another reason could be the complexity in defining and collecting data for indicators that precisely capture these concepts. For example, measuring efficiency requires controlling for variations in quality of care, which is often not linked to cost data [75]. Moreover, measuring the achievement of health system goals, such as responsiveness, improved health, and efficiency, requires isolating the effects of determinants that fall outside the health system, which remains a challenge, and is still a work in progress [76].

The lack of attention to ‘Health information systems’ is surprising, given their central role for generating data that can be used to describe and steer health systems [71, 77]. We believe that this topic deserves more attention in the revision of existing or the development of new templates. Likewise, ‘governance’ is not always covered in existing templates, despite its importance for health systems' performance and resilience [78]. Potentially, the systematic description and assessment of governance could be improved through the incorporation of governance indicators that have been developed by various organizations [79]. Similarly, templates could be improved with regard to their approach to describing and measuring ‘efficiency’ and ‘responsiveness’ based on several analytical frameworks and indicators that have been developed [7, 9–11, 73, 75, 80–85].

### Limitations

This study has several limitations. First, our overview of templates’ contents is omitting details and nuances, given that it was impossible to describe the actual questions and indicators of each template. Second, the choice to define and analyse templates based on the WHO’s health systems framework (2007) [22] may have biased our results, as we excluded from further analyses all documents that did not cover most building blocks. There are many different frameworks for the analysis of health systems, in part because a society’s cultural values influence how policy-makers, researchers or other stakeholders conceptualize and measure the performance of health system goals [48]. The choice of a certain framework certainly influences the outcomes measured—i.e., the ‘goals’ of the health system. Other frameworks or approaches would have yielded different results. Despite the disadvantages of the WHO framework, it is commonly used by policy-makers and researchers in HICs and LMICs. Therefore, we believe that it was the most suitable framework for our purpose. Finally, three of the authors (AM, BR and EvG) are authors of two of the templates, which may have influenced the conceptualization of a “template” and the choice of defining criteria. However, they tried to be as impartial as possible in this work, and the other authors balanced this potential bias.

## Conclusions

Standardized reports of health systems’ characteristics and systematic assessments of their performance can support health systems strengthening. Templates should guide authors to describe the structure and functioning of any health system, while allowing for flexibility to account for context. Our review of templates shows that some health system building blocks (i.e. ‘governance’ and ‘health information systems’) and certain health system goals (i.e. ‘responsiveness’ and ‘efficiency’) are missing in several templates. A comprehensive health systems analysis and comparison requires a combination of quantitative indicators complemented with qualitative information to build a more holistic picture. The implications of our findings are that (1) policy-makers should demand systematic template-guided analyses of their health systems to underpin national health policies, strategies, and plans; (2) organisations developing templates should be inspired to learn from approaches of other templates at describing and assessing health systems and consider the incorporation of more qualitative information; and (3) researchers should strive to fill important knowledge gaps on indicators for health systems descriptions and assessments, as well as exploring how templates can be further improved to better achieve the goal of supporting policy-makers in strengthening health systems worldwide.

### Supplementary Information

Supplementary Materials 1.

## Data Availability

Data is publicly available.

## Abbreviations

CEF-AHI: Common evaluation framework for the African Health Initiative
CHSSP: Country Health Systems and Service Profile
CMWF: Commonwealth Fund
EMRO: WHO’s Eastern Mediterranean Region
EU: European Union
HIC: High Income Countries
HiT: Health Systems in Transition
HSAA: Health System Assessment Approach
HSCP: Health Systems Country Profiles
HSCS: Health systems Characteristics survey
HSiAI: Health Systems in Action insights
LMIC: Low and Middle-income countries
MFUHA: Monitoring Framework for Universal Health in the Americas
NCD: Non-communicable disease
OECD: The Organisation for Economic Co-operation and Development
PAHO: Pan American Health Organization
QUAL: Qualitative
QUAN: Quantitative
SoHEU: State of Health in the EU
UHC: Universal Health Coverage
UHC-MF: Universal Health Coverage Monitoring Framework
USAID: United States Agency for International Development
WHO: World Health Organization
WHOLIS: WHO Library and Digital Information Networks
WHO-MBB: WHO Monitoring the Building Blocks

## Footnotes

1 For example, in some databases, the Boolean operators did not work (ELDIS) or the search field was limited to a certain number of characters (Google scholar).
