# Supplementary material for: Health system description and assessment: a scoping review of templates for systematic analyses
Source: Health Res Policy Syst. 2024 Jul 11;22:82. doi: 10.1186/s12961-024-01166-y (PMC11238392; doi:10.1186/s12961-024-01166-y)
Supplement: Supplementary file 1 — Supplementary Materials 1. [file 12961_2024_1166_MOESM1_ESM.docx]

# Electronic supplementary material

## Supplement 1: List of data sources

1. Grey Literature Databases: OpenGrey, ELDIS, WHOLIS, Google Scholar
2. Search Engines: Google, Bing AI, ELICIT
3. Targeted Websites:

| **Organization** | **Website** |
| --- | --- |
| WHO | https://www.who.int/ |
| WHO Regional Office: African region | <https://www.afro.who.int/> |
| WHO Regional Office: Americas | <https://www.paho.org/en> |
| WHO Regional Office: South East Asia | <https://www.who.int/southeastasia> |
| WHO Regional Office: Europe | https://www.who.int/europe/home?v=welcome |
| WHO Regional Office: Eastern Mediterranean | <https://www.emro.who.int/index.html> |
| WHO Regional Office: Western Pacific | https://www.who.int/westernpacific/ |
| African Health Observatory Platform on Health Systems and Policies (AHOP) | [AHOP – African Health Observatory Platform (AHOP) (who.int)](https://ahop.aho.afro.who.int/) |
| Eastern Mediterranean Health Observatory (RHO) | [Home \| EMRO Regional Health Observatory (who.int)](https://rho.emro.who.int/index.php/) |
| European Observatory on Health Systems and Policies | [European Observatory on Health Systems and Policies (who.int)](https://eurohealthobservatory.who.int/) |
| Asia Pacific Observatory on Health Systems and Policies (SEARO) | [World Health Organization, South-East Asia Regional Office (who.int)](http://www.searo.who.int/entity/asia_pacific_observatory/en/) |
| UNICEF | https://www.unicef.org/ |
| UNAIDS | <https://www.unaids.org/en> |
| WORLDBANK | https://www.worldbank.org/en/home |
| Commonwealth Fund | https://www.commonwealthfund.org/ |
| Afrobarometer | [About – Afrobarometer](https://www.afrobarometer.org/about/) |
| The Organisation for Economic Cooperation and Development | <https://www.oecd.org/> |

1. Interviewing Experts: authors of the scoping review

## Supplement 2: Full electronic search: search engines, strings, hits and number of hits included for screening

| **Search engine** | **Search string** | **Number of Hits** | **Number of hits included for screening** | **Date of search** |
| --- | --- | --- | --- | --- |
| Bing AI https://www.bing.com/?/ai | Please show me templates from international organizations, that describe or assess or compare health systems of countries. | 3 | 3 | 25.07.2023 |
| Bing AI https://www.bing.com/?/ai | Can you show me reports that describe health systems and health in countries? | 3 | 3 | 25.07.2023 |
| https://elicit.org/ | Please show me templates from international organizations, that describe or assess or compare health systems of countries. | no number of search result shown, first 100 hits screened | 21 | 25.07.2023 |
| https://elicit.org/ | Can you show me reports that describe health systems and health in countries? | no number of search result shown, first 100 hits screened | 7 | 25.07.2023 |
| Google | 1.(Report OR assessment OR tool OR manual OR guideline OR guide OR guidance OR outline OR evaluation OR survey OR profile OR account OR monitoring OR description OR instruction) AND (“health system” OR “health sector” OR “health care system” OR “health care performance” OR “health”)  2. (Report OR assessment OR tool OR manual OR guideline OR guide OR guidance OR outline OR evaluation OR survey OR profile OR account OR monitoring OR description OR instruction) AND (“health system” OR “health sector” OR “health care system” OR “health care performance” OR “health”) AND (cross-country OR international OR comparison OR similarity OR difference) | 1. 9.880.000.000 (only first 100 screened)  2. 6.030.000.000 (only first 100 hits screened) | 1. 12 2. 5 | 03.12.2023 |
| OpenGrey | 1. (Report OR assessment OR tool OR manual OR guideline OR guide OR guidance OR outline OR evaluation OR survey OR profile OR account OR monitoring OR description OR instruction) AND (“health system” OR “health sector” OR “health care system” OR “health care performance” OR “health”) Filter: Life sciences, medicine and health care; Health Sciences; any field 2. (Report OR assessment OR tool OR manual OR guideline OR guide OR guidance OR outline OR evaluation OR survey OR profile OR account OR monitoring OR description OR instruction) AND (“health system” OR “health sector” OR “health care system” OR “health care performance” OR “health”) AND (cross-country OR international OR comparison OR similarity OR difference) Filter: Life sciences, medicine and health care; Health Sciences; any field | 1. 97 2. 13 | 1. 0 2. 0 | 22.07.2023 |
| ELDIS | 1. Health Monitor Filter: Focus Topic: Health Systems 2. Health Survey Filter: Focus Topic: Health Systems 3. Health report Filter: Focus Topic: Health Systems 4. Health Profile Filter: Focus Topic: Health Systems 5. Health evaluation Filter: Focus Topic: Health Systems 6. Health description comparison Filter: Focus Topic: Health Systems 7. Health account Filter: Focus Topic: Health Systems 8. Health performance Filter: Focus Topic: Health Systems | 1. 314 2. 178 3. 702 4. 38 5. 293 6. 30 7. 314 8. 280 | 1. 7 2. 3 3. 22 4. 3 5. 4 6. 2 7. 10 8. 8 | 22./23.07.2023 |
| WHOLIS | 1. (Report OR assessment OR tool OR manual OR guideline OR guide OR guidance OR outline OR evaluation OR survey OR profile OR account OR monitoring OR description OR instruction) AND (health system OR health sector OR health care system OR health care performance OR health) Filter: Search in: keywords; Publication date range: 2000-2023 2. (Report OR assessment OR tool OR manual OR guideline OR guide OR guidance OR outline OR evaluation OR survey OR profile OR account OR monitoring OR description OR instruction) AND (health system OR health sector OR health care system OR health care performance OR health) AND (cross-country OR international OR comparison OR similarity OR difference) Filter: Search in: keywords; Publication date range: 2000-2023 | 1. 2585 2. 494 | 1. 43 2. 5 | 03.12.2023 |
| Google Scholar | 1. (Report OR assessment OR tool OR manual OR guideline OR guide OR guidance OR outline OR evaluation OR survey OR profile OR account OR monitoring OR description OR instruction) (health system OR health sector OR health care system) Filter: time range: 2000-2023 2. (Report OR assessment OR tool OR manual OR guideline OR guide OR guidance OR outline OR evaluation OR survey OR profile OR account OR monitoring OR description OR instruction) (health care performance OR health) Filter: time range: 2000-2023 3. (Report OR assessment OR tool OR manual OR guideline OR guide OR guidance OR outline OR evaluation OR survey OR profile OR account OR monitoring OR description OR instruction) (health care performance OR health) (international OR comparison) Filter: time range: 2000-2023 | 1. 2280 (only first 100 hits screened) 2. 220 000 (only first 100 hits screened) 3. 17 800 | 1. 10  2. 0  3. 0 | 25.07.2023 |
| Experts |  | 14 | 14 |  |

## Supplement 3: Documents that did not meet all the criteria to be considered a “template”

| **Documents that are limited to specific topics, and are not considered ‘templates’** | | | | | |  |
| --- | --- | --- | --- | --- | --- | --- |
| **Document** | **Framework** | **List of indicators** | **Instructions for authors** | **System design/structure** | **System performance** | **Covers at least four of the six functions of WHO’s building blocks** |
| 1. WHO Health Systems Performance Assessment - [Debates, Methods and Empiricism](http://whqlibdoc.who.int/publications/2003/9241562455.pdf?ua=1), 2003 [39] | √ | √ | √ (minimal) | √ | √ [only methods] |  |
| 1. USAID Using Data Analytics to [Monitor Health Provider Payment Systems](https://www.jointlearningnetwork.org/resources/data-analytics-for-monitoring-provider-payment-toolkit/), 2016 [77] | √ | √ | √ | √ | √ | [payment mechanisms] |
| 1. USAID Essential Packages of Health Services [benefits basket] in [24 Countries](https://www.hfgproject.org/ephs-cross-country-analysis/) + [Country Reports](https://www.hfgproject.org/ephs-epcmd-country-snapshots-series/) [78] | √ | √ | √ (implicit) | √ [benefits basket] |  |  |
| 1. WHO - [Assessing the National Health Information System](https://apps.who.int/iris/handle/10665/43932) - An Assessment Tool [79] | √ | √ | √ |  | √ [HIS] |  |
| 1. OECD Health Care Quality [Framework](https://www.oecd-ilibrary.org/social-issues-migration-health/health-care-quality-indicators-project_440134737301) [80] | √ | √ | √ (implicit) |  | √ | [quality of care] |
| 1. OECD [Country Reviews](http://www.oecd.org/health/health-systems/health-care-quality-reviews.htm) of National Health Care Quality[81] | √ | √ | √ (implicit) | √ | √ | [only quality of care] |
| 1. Papanicolas, I. and Smith, P. Policy Brief: [Health system performance comparison](http://eprints.lse.ac.uk/54802/): an agenda for policy, information and research, 2013 [40] | √ | √ | √ |  | √ [methods] |  |
| 1. WHO and the World Bank- Tracking universal health [coverage](https://www.who.int/healthinfo/universal_health_coverage/report/2015/en/), 2015 and 2017 [82] (based on WHO World health [survey](https://apps.who.int/healthinfo/systems/surveydata/index.php/catalog/whs/about) (2002-2004) | √ | √ |  | √ (implicit) | √ | [Coverage and financial protection] |
| 1. Joumard, I., Andre, C. and Nicq, C. (2010) Health care systems: efficiency and institutions (OECD Economics Department [Working Paper](https://www.oecd-ilibrary.org/health-care-systems_5kmfp51f5f9t.pdf), no. 769).[83] | √ (implicit) | √ | √ |  | √ | [Efficiency] |
| 1. Primary Health Care Performance Initiative ([PHCPI](https://improvingphc.org/sites/default/files/PHC-Progression%20Model%202019-04-04_FINAL.pdf)), 2019[84] | √ | √ | √ |  | √ | [primary care] |
| 1. [PHAMEU](https://www.euro.who.int/en/about-us/partners/observatory/publications/studies/building-primary-care-in-a-changing-europe-2015) (Primary Health Care Activity Monitor for Europe)[85] | √ | √ | √ | √ | √ | [primary care] |
| 1. [DISMEVAL](https://www.rand.org/pubs/technical_reports/TR1226.html) - Developing and validating disease management evaluation methods for European healthcare systems + country reports[86] | √ | √ | (qualitative data from interviews) |  | √ | [specific diseases] |
| 1. Monitoring mental healthcare on a system level: Country profiles and status from EU countries [87] | √ | √ | √ | √ [mental health] | √ | [mental health] |
| 1. WHO Service availability and readiness assessment ([SARA](https://www.who.int/healthinfo/systems/SARA_OverviewPresentation.pdf?ua=1)), 2015 [88] | √ | √ | √ |  | √ | [Service Availability and Readiness] |
| 1. European Commission - [tools and methodologies for assessing the performance of primary care](https://ec.europa.eu/health/sites/health/files/expert_panel/docs/opinion_primarycare_performance_en.pdf) [89] | √ | √ | √ | √ (minimal) | √ | [primary care] |
| 1. OECD [health at a glance](http://www.oecd.org/health/health-systems/health-at-a-glance-19991312.htm), (HaG) 2019 [90], [91] | √ | √ |  | √ (minimal) | √ |  |
| 1. [Monitoring health and health system performance in the Eastern Mediterranean Region: Core indicators and indicators on the health-related Sustainable Development Goals](https://applications.emro.who.int/docs/WHOEMHST247E-eng.pdf), 2021. Cairo: WHO Regional Office for the Eastern Mediterranean; 2022. [92] | √ (implicit) | √ |  | √ | √ |  |
| 1. [Health and well-being profile of the Eastern Mediterranean Region: an overview of the health situation in the Region and its countries in 2019.](https://rho.emro.who.int/Article/health-and-well-being-profile-of-the-eastern-mediterranean-region) Cairo: WHO Regional Office for the Eastern Mediterranean; 2020. [93] | √ (implicit) |  | √ | √ | √ |  |
| 1. [Atlas of African Health Statistics 2022: Health situation analysis of the WHO African Region](https://aho.afro.who.int/atlas/af). Brazzaville: WHO Regional Office for Africa; 2022. [94] | √ | √ |  |  | √ |  |
| 1. [Health Care in Central Asia;](https://www.google.de/url?sa=i&rct=j&q=&esrc=s&source=web&cd=&cad=rja&uact=8&ved=0CAIQw7AJahcKEwjw5MTZ96SAAxUAAAAAHQAAAAAQAg&url=https%3A%2F%2Feurohealthobservatory.who.int%2Fdocs%2Flibrariesprovider3%2Fstudies---external%2Fhealth-care-in-central-asia.pdf%3Fsfvrsn%3Dd2ba9265_3%26download%3Dtrue&psig=AOvVaw0RfBWUqMlfVmZ3Ki7vTsui&ust=1690205324169345&opi=89978449) World Health Organisation, 2002 [95] |  | √ |  | √ |  |  |
| 1. [Health in the Americas Country profiles](https://hia.paho.org/en). PAHO [96] |  | √ |  |  | √ |  |
| 1. [Health system briefs, EMRO](https://rho.emro.who.int/index.php/health-system-profiles) [97] |  | √ |  | √ | √ |  |
| 1. [„Mirror, Mirror“, Commonwealth](https://www.commonwealthfund.org/publications/fund-reports/2021/aug/mirror-mirror-2021-reflecting-poorly) [98] | √ |  | √ |  | √ |  |
| 1. [Monitoring Health for the Sustainable Development Goals (SDGs).](https://www.who.int/docs/default-source/gho-documents/world-health-statistic-reports/world-heatlth-statistics-2016.pdf) World Health Organization. (2016). World Health Statistics [99] |  | √ |  |  | √ |  |
| 1. [The African regional health report: the health of the people](https://www.afro.who.int/sites/default/files/2017-06/african_regional_health_report2006_0.pdf); Regional Office for Africa, World Health Organisation, 2006 [100] |  | √ |  | √ | √ |  |
| 1. [The health-related Sustainable Development Goals](https://www.who.int/westernpacific/publications-detail/9789290619512): progress report of the western pacific region. 2020 [101] | √ |  |  |  | √ |  |

Notes: 1. These are the latest versions of the templates at time of search. By time of publication of this work, newer versions are available. Newer or older versions might have different contents, topics, indicators. 2. (minimal) = the element exists in the document, but only to a very limited extent. 3. Topics in brackets, e.g. [mental health] are the focus of the documents not considered templates.
